# Stakeholder Perceptions and Context of the Implementation of Performance-Based Financing in District Hospitals in Mali

**DOI:** 10.15171/ijhpm.2019.45

**Published:** 2019-06-30

**Authors:** Tony Zitti, Lara Gautier, Abdourahmane Coulibaly, Valéry Ridde

**Affiliations:** ^1^CEPED (UMR 196), Institut de Recherche pour le Développement, ERL INSERM SAGESUD, École doctorale Pierre Louis de santé publique, Université Sorbonne Paris Cité, Paris, France.; ^2^Miseli Research NGO, Bamako, Mali.; ^3^Department of Social and Preventive Medicine, University of Montreal, Montreal, QC, Canada.; ^4^Public Health Research Institute, University of Montreal, Montreal, QC, Canada.; ^5^CESSMA (UMR 245), Institut de Recherche pour le Développement, Université Sorbonne Paris Cité, Paris, France.; ^6^Faculty of Medicine and Odonto-Stomatology, Université des Sciences, des Techniques et des Technologies, Bamako, Mali.; ^7^CEPED (UMR 196), Institut de Recherche pour le Développement, ERL INSERM SAGESUD, Université Sorbonne Paris Cité, Paris, France.

**Keywords:** Performance-Based Financing, Mali, Implementation, CFIR, District Hospitals

## Abstract

**Background:** To improve the performance of the healthcare system, Mali’s government implemented a pilot project of performance-based financing (PBF) in the field of reproductive health. It was established in the Koulikoro region. This research analyses the process of implementing PBF at district hospital (DH) level, something which has rarely been done in Africa.

**Methods:** This qualitative research is based on a multiple, explanatory, and contrasting case study with nested levels of analysis. It covered three of the 10 DHs in the Koulikoro region. We conducted 36 interviews: 12 per DH with council of circle’s members (2) and health personnel (10). We also conducted 24 non-participant observation sessions, 16 informal interviews, and performed a literature review. We performed data analysis using the Consolidated Framework for Implementation Research (CFIR).

**Results:** Stakeholders perceived the PBF pilot project as a vertical intervention from outside that focused solely on reproductive health. Local actors were not involved in the design of the PBF model. Several difficulties regarding the quality of its design and implementation were highlighted: too short duration of the intervention (8 months), choice and insufficient number of indicators according to the priority of the donors, and impossibility of making changes to the model during its implementation. All health workers adhered to the principles of PBF intervention. Except for members of the district health management team (DHMT) involved in the implementation, respondents only had partial knowledge of the PBF intervention. The implementation of PBF appeared to be easier in District 3 Hospital compared to District 1 and District 2 because it benefited from a pre-pilot project and had good leadership.

**Conclusion:** The PBF programme offered an opportunity to improve the quality of care provided to the population through the motivation of health personnel in Mali. However, several obstacles were observed during the implementation of the PBF pilot project in DHs. When designing and implementing PBF in DHs, it is necessary to consider factors that can influence the implementation of a complex intervention.

## Background

Performance-based financing (PBF) is a mechanism by which health facilities are paid on the basis of their performance, which is measured by the quantity and quality of services they provide.^1^ This practice would increase the productivity and quality of healthcare benefits available to people.^2,3^ PBF is expanding rapidly in low- and middle-income countries,^4^ and gaining interest from governments and development agencies.^5,6^ However, the results of several systematic reviews are mixed with respect to the effects of PBF on the use and the quality of health services.^7-10^ Studies point to a gap between expectations and achievements in the experimenting PBF.^11-14^ In Benin, Antony et al^14^ emphasised the complexity and high cost of qualitative, quantitative, and community verification processes. In Nigeria, Ogundeji et al^12^ showed that the delay and lack of communication in the payment of PBF financial rewards have led to uncertainty and mistrust among health workers about PBF, causing a negative impact on health workers motivation. In Cameroon, a study by De Allegri et al^15^ revealed that the delay due to the payment of PBF bonus payments did not allow the health centre to have effective management autonomy, to execute their plans, and to cover the costs of taking care of the very poor. The majority of articles on the implementation of the PBF in Africa^11,12,16^ either featured a wide range of health facilities (dispensaries, health posts, health centres, and hospitals) or just focused on primary health centres.

Thus, the particular context prevailing in district hospitals (DHs) is not prominently or systematically featured in articles studying the implementation of PBF in Africa. In Malawi, Lohmann et al^16^ showed that in community-level health facilities, individual financial incentives were distributed to all staff at the centre, while in the DHs, priority was given to health workers working in maternity wards. In Rwanda, Paul’s study^17^ explained that distribution of bonus payments between health workers in DHs was not done on the basis of individual performance evaluations, but rather on the performance of health units or hospitals. In addition, this case study in Rwanda demonstrated that the performance of health workers in DHs was dependent on several factors related to the work environment (equipment availability, leadership, and communication), and health workers’ characteristics (norms and values in relation to work). Clearly, a problem of transparency and lack of information arose during the implementation of PBF, which in some cases impacted the motivation of DH health workers. In Burundi, a qualitative DH level study highlighted that stakeholders’ views and the context of implementation need to be taken into account from the design and during the implementation of the PBF.^18^ In Burkina Faso, a study from Bodson et al^19^ showed that PBF was more faithfully implemented at the level of primary health centres compared to hospitals. However, this study did not concern the analysis of the contextual factors that could explain these results. Our article aims to fill a research gap in the existing literature, by analysing the process of implementation of PBF at the DH level of Koulikoro region in Mali. The following research question guides this study: how is PBF implemented in DHs? We aim to understand the problems related to the design of the PBF intervention model and the influence of the local context; and to highlight the specific local norms and values guiding the implementation of PBF at the DH level.

### History of Performance-Based Financing in Mali 

From February 2012 to December 2013, a pre-pilot PBF project was initiated in the Koulikoro region, specifically in the districts of Dioïla, Fana, and Banamba. The project was implemented following a partnership between the Ministry of Health and Public Hygiene (Ministère de la Santé et de l’Hygiène Publique [MSHP]), the Netherlands Development Organization (SNV), and the Royal Tropical Institute (KIT). An evaluation of the project was carried out in May 2014 by consultants directly involved in designing and implementing the intervention.^20^ In their report, the authors claimed to identify an increase in the use of health facilities for certain services concerning maternal health and an improvement in the quality of care. However, an independent study showed that the introduction and removal of PBF had no effect on the use of maternal and child health services.^21^ In addition, another study showed that the degree of sustainability for this intervention was weak.^22^

Subsequently, Mali’s government, as part of a large World Bank-funded Strengthening Reproductive Health Project (SRHP), expanded the PBF initiative to all ten health districts from the Koulikoro region.^23^ SRHP was intended to support Mali’s efforts to strengthen its health system in a number of ways: (*i*) strengthening the supply and quality of reproductive health services; (*ii*) increasing demand for reproductive health services; and (*iii*) social responsibility, project management, and monitoring and evaluation.^24^ The PBF pilot project is one of the sub-components of SRHP. According to its designers, the goal of the PBF strategy is to increase the use of quality reproductive health services by increasing the motivation and accountability of service providers to achieve results. PBF involved a total of 205 Community Health Centres (Centres de santé communautaires [CSCom]) and 10 DHs. Implementation of the PBF component of the SRHP started in July 2016 for a period of 8 months.^24^

### Architecture of the Performance-Based Financing Pilot in Mali 

The actors who implemented the PBF pilot project decided that it embraces the architecture of the health system without introducing new structures. At the central level, an interministerial steering committee was set up to monitor the implementation of the SRHP. The SRHP was coordinated by the Project Coordination Unit, with which the MSHP had signed a contract. A contract was also signed between the Project Coordination Unit and a multi-stakeholder consortium. The consortium, which included 2 Dutch organisations (KIT and Cordaid) and a local company (Clinique de Gestion et d’Innovation des Connaissances [CGIC]), was tasked with project operationalisation. The consortium acted as a contracting and verification agency. To assist local actors in the implementation of PBF, 10 technical assistant experts in PBF were recruited and assigned to each DH level. Five of these technical assistant experts were international experts from other African countries, and 5 were Mali nationals.

At the local level, a PBF contract was signed between the KIT-Cordaid-CGIC Consortium and the circle council (ie, the administrative body responsible for managing the circles) overseeing the DHs. Circles are sub-district administrations that gather several municipalities, endowed with a legal personality and benefiting from financial autonomy. Subsequently, circle councils signed a contract with DHs for the implementation of PBF. DHs developed results plans quarterly, which identified the main barriers to improving health indicators in geographic areas. It suggested solutions to solve health system and healthcare issues, and the means to implement those solutions. The development of the results plans also involved health workers and the participation of members from the circle council. As a regulator, the role of the Regional Health Directorate (Direction régionale de la santé [DRS]) was to ensure the respect of quality standards in DHs. Three quantitative indicators were chosen for PBF in DHs (Table S1, Supplementary file 1). Amounts for indicators paid under PBF were decided before beginning implementation. DRS members checked DH records monthly to verify the total number of services offered. Quality indicators were checked quarterly by members of the DRS. Verification of qualitative indicators resulted in the attribution of a technical quality score are expressed as a percentage (Table S2, Supplementary file 1). In DHs, a maximum of 60% of the PBF subsidies were planned to reward individual motivation of health workers, and a minimum of 40% was to be spent on equipment. Once quantitative and qualitative indicators had been checked, the PBF subsidies were channelled into bank accounts of DHs. Counter-verification of user performance was done by local non-governmental organisations (NGOs).

## Methods

### Setting of the Study 

The study took place in Mali, in the Koulikoro region. In this country, the health system pyramid has 3 levels (Figure S1, Supplementary file 1). Table S3 outlines socio-demographic and health characteristics of Mali and the Koulikoro region.

### Conceptual Framework 

Data collection and process analysis were carried out using the Consolidated Framework for Implementation Research (CFIR).^25^ According to this framework, 5 dimensions should be studied to understand the implementation of a health intervention (Figure): (*i*) Characteristics of PBF intervention, (*ii*) Outer Setting to DH, (*iii*) Inner Setting to DH, (*iv*) Characteristics of Individuals, and (*v*) Process (of PBF). Each of these dimensions includes several constructs. Thanks to a preliminary analysis of the CFIR conceptual framework by the research team, we were able to classify the selected constructs and their descriptions as well as the constructs not retained and their justification (see Table S4, Supplementary file 1).

**Figure:**
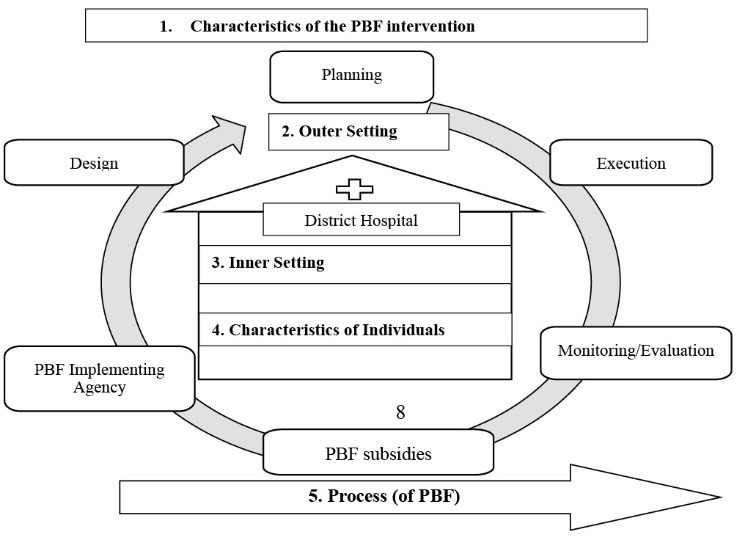
Conceptual Framework, Adapted From Damschroder et al.^25^
Abbreviation: PBF, performance-based financing.

### Research Strategy 

We adopted a qualitative approach, based on a set of explanatory and contrasting multiple case studies with nested levels of analysis^26^ corresponding to DHs and participants to the implementation of PBF. The conceptual framework outlined above (CFIR) guides our case studies. As per Yin’s 5 components of a case study, our study used: (1) a research question: how is PBF implemented in DHs?; (2) one main proposition in relation to the research question: the characteristics of the PBF intervention, the outer and inner setting of DHs, the characteristics of individuals, and the processes embedded in the intervention affect the implementation of PBF; (3) units of analysis (detailed in the paragraph below); (4) logic linking of the data to the propositions; and (5) the following criteria for interpreting the findings: CFIR’s 5 dimensions (ie, characteristics of the PBF intervention, outer setting of DH, inner setting of DH, characteristics of individuals, and PBF process) guiding data collection (eg, interview guides) and analytical approach.

We chose 3 of the 10 DHs in the Koulikoro region. The characteristics of the 3 health districts and their DH are summarised in Table S5, Supplementary file 1. These figures take into account our resource constraints, but also feature an adequate representation of the diversity of contextual situations conducive to the analytical generalisation process specific to case.^26^ Our study is part of a wider research programme entitled: “*Results-based financing for equitable access to maternal and child health care in Mali and Burkina Faso.”* The cases were those identified for this research programme.

Several districts were removed from the eligible cases because they did not represent the regular context of the health system that the intervention aims to improve (strong interventions by several NGOs), or are not accessible for security reasons. District 1 was selected for its urban character and the presence of a medical assistance scheme (Régime d’assistance médicale [RAMED]). In Mali, RAMED provides medical care for the poor and other vulnerable populations. District 2 was chosen for featuring an articulation of PBF with a community-based insurance was envisaged. This was intended to assess the extent to which PBF could serve as a basis for the accreditation process of health facilities, a process that predates the development of social protection systems (compulsory health insurance, community-based insurance, and RAMED). Finally, we selected District 3, a landlocked and agricultural area in which a community experiment to identify the poorest was also tested. Of the 3 districts selected, only District 3 experienced the PBF pilot phase.

### Sampling of Participants 

Purposeful sampling was used to select participants, so as to ensure external diversification. This selection makes it possible to contrast the different points of view of actors who occupy different positions within a group, in order to have an overall analysis that can be generalised.^27^ For each district, it was necessary to identify the different categories of actors involved in the implementation of PBF at the level of the 3 DHs. Different stakeholder profiles (Table 1) were selected to compare points of view.^27^ Participants were recruited based on their availability to answer interview questions.

**Table 1:** People Surveyed at the Level of the 3 DHs

**People Surveyed**	**DH 1**	**DH 2**	**DH 3**	**Total**
Circle council members	2	2	2	**6**
District health managers	1	1	1	**3**
Medical doctors	1	1	1	**3**
PBF focal points	1	1	1	**3**
Agents in charge of health information systems	1	1	1	**3**
Nurses	2	2	2	**6**
Obstetrician nurses	2	2	2	**6**
Pharmacist/drug’s managers	1	1	1	**3**
Agent in charge of hygiene/sanitation	1	1	1	**3**
**Total**	**12**	**12**	**12**	**36**

Abbreviations: PBF, performance-based financing; DH, district hospital.

### Tools, Techniques, and Data Collection 

The research was conducted from December 2016 to January 2017. We stayed for 12 days in each of the 3 DHs. Actors’ perceptions and practices was the centre of the interview questions. Three semi-structured interview guides were prepared for the District Health Manager (Médecin chef de District [MCD]), staff (medical doctors, nurses, and other health workers and staff), and circle council members. The contents of the 3 guides have been adapted with the selected CFIR constructs.

We conducted 36 semi-structured interviews. We also conducted 24 non-participant observation sessions as well as 16 informal interviews with 2 caretakers, 5 physicians, 1 pharmacist, 1 health information officer, 1 social development officer, 2 nurses, 2 interns, 1 midwife, 1 nurse woman, and 1 hygienist. Personal notes taken during non-participant observations and informal interviews were recorded in a journal. In the non-participant observation sessions, we focused on the following topics: (*i*) work environment (hygiene in hospital outbuildings, treatment rooms, waiting areas, washrooms, etc); (*ii*) technical tools in DHs (ie, availability and filling of clinical records, attendance books); and (*iii*) work performance (ie, quality of reception, quality of orientation, guard system).

### Data Processing and Analysis

Data processing was done iteratively. All interviews and notes written were classified by site. Research assistants transcribed verbatim all the recordings. The transcribed data was reviewed and coded using a codebook derived from our theoretical framework.^28^ We coded the data using the QDA Miner Lite software. Based on these codes, we conducted data analysis using the CFIR. This method allowed for an analytical approach that followed a deductive-inductive logic, based on the CFIR dimensions, and allowed empirical themes to emerge that may be relevant to better understand the implementation of PBF in Mali. Our results are presented using the 5 dimensions of the CFIR framework, following a comprehensive and logical flow as recommended by a conceptor of the CFIR.^25^

## Results

Our results feature a general analysis of the implementation of PBF in DHs in Mali. Very few empirical differences appeared between the cases: only significant differences were highlighted. In this section, the themes match the dimension of the CFIR. CFIR sub-constructs are shown in brackets.

### Characteristics of the Performance-Based Financing Intervention 

The majority of the actors were not aware of the source of financing for the PBF pilot intervention (*Intervention Source*):

‘‘*I think maybe it’s the partners (who brought in PBF), but I have no idea. I have not received any information about that’’ [Nurse, Case 2].*

Only respondents among district health management team (DHMT) members involved in the implementation knew that the World Bank funded the PBF intervention. Respondents believed that PBF provided a better return on investment as compared to another existing project (*Relative Advantage*). However, they perceived the project as being too short (8 months) because it only allowed for one payment cycle in a single quarter:

“*The duration of the implementation is a factor of discouragement, because the change of system requires a [comprehensive] support system; it is not in 8 months that it can do it, especially where there’s staff turnover”* [Agent in charge of hygiene/sanitation, Case 3].

Furthermore, respondents perceived PBF as a vertical program, ie, only focusing on reproductive health and not allowing involvement of all DH care services (*Complexity*). Moreover, they considered that choosing 3 quantitative indicators was insufficient (*Design, Quality, and Packaging*):

“*When you look at the documents that talk about PBF internationally, you are told that for a district hospital [DH] it takes for example twelve quantity indicators, we are at three”* [District Health Manager, Case 3].

Several actors perceived this PBF intervention as a very complex system, ie, the multitude of procedures and approaches made its implementation at the local level very slow (*Complexity*). In addition, it was not possible to adapt the intervention as deficiencies were observed (*Adaptability*). PBF was an original action for the majority of DH 1 and 2 staff, but not for DH 3 staff who had already experienced it (*Trialability*). Information on the implementation interest and effectiveness of the pre-pilot PBF project was provided during PBF training. Almost all respondents believed that PBF had already been proven during the pre-pilot phase. They believed that the pre-pilot project had resulted in individual benefits and improved both indicators and the quality of care provided (*Evidence Strength and Quality*):

*“The people who participated in the PBF pre-pilot project say that it brought them money. In addition it allowed them to supply their structure”* [Medical Doctor, Case 1].

### Outer Setting to Health Facilities 

The PBF reform is featured in the Decennial Health and Social Development Programme III (Programme Décennal de Développement Sanitaire et Social) period 2014-2023 as an innovative approach that can improve the healthcare quality (*External Policy and Incentives*). Informants reported that indicators selected matched donors’ priorities. Local actors were not involved in the design process (choice indicators, etc) of PBF. Yet, informants considered that quantitative indicators of PBF chosen at the DH level concerned maternal mortality issues of national priority. In addition, several actors argued that some relevant indicators such as curative consultation, fourth prenatal consultation, family planning, and the third post-natal consultation should have been taken into account (*Patient Needs and Resources*). To facilitate the implementation of PBF, DHs collaborated with external organisations like circle councils, the implementing agency (ie, the consortium including the technical assistant experts in PBF), and CSCom (*Cosmopolitanism*).

### Inner Setting to Health Facilities 

The availability of local qualified personnel and equipment were factors that reportedly facilitated the implementation of PBF (*Structural Characteristics*). However, several problems experienced in DHs prevented adequate implementation of PBF (Table 2), such as malfunctioning management bodies (ie, management board and DHMTs) or staff mobilisation for meetings. Communication channels and tools were defective in the vast majority of DHs (*Networks and Communications*). In addition, we noted a problem of motivation for health workers: they did not have a performance culture in their work (*Culture*). PBF was implemented in a difficult context prevailing in DHs (*Implementation Climate/Tension for Change*).

**Table 2:** Summary of the Inner Setting That Prevailed Before the Implementation of PBF

**Dimensions**	**Problems Experienced**
Care delivery	- Deficit of the technical platform - Staff instability - Non-compliance with standards and procedures - Inadequate retraining of health workers
Management-body (management board and DHMT)	- Malfunction of management bodies - Only MCD and managers were involved in financial management - Non-transparent management - Trust between staff and managers
Leadership of the MCD	- Lack of leadership capacity in DH 1 - Authoritarian leadership capacity in DH 2 - Good leadership capacity in DH 3
Hygiene and sanitation	- Presence of a sanitation hygiene unit - The hygiene of the premises and the courtyard was better in DH 2 and 3 than in DH 1
Patient reception and orientation	- The reception of patients and their orientation was well done, and the name of each unit was placed on the buildings in French and local language (*Bambara*) in DH 3 - In DH 1 and 2, there was a serious problem with patient referral, as care units were not identified
Guard system	- A 7-day/7-day, 24-hour on-call system existed in all three DHs - In DH 1 a premium was paid to the DH guard, unlike in DH 2 and 3
Drug supply system for the health district	- Well-functioning

Abbreviations: PBF, performance-based financing; DH, district hospital; MCD, District Health Manager (Médecin chef de District); DHMT, district health management team.

Before the start of PBF implementation, all MCDs participated in a 2-week training in Benin in May 2016. A *PBF focal point*, (ie, a person assisting some MCD with PBF implementation), was appointed in each structure by the MCD. The decision to appoint a PBF focal point was entirely at the discretion of each MCD. The MCD and the PBF Focal Point also participated in several other trainings at national and regional levels (*Readiness for Implementation/Access to Knowledge and Information*). Two training sessions were conducted in each DH by the PBF focal point with the assistance of the technical assistant experts in PBF provided by the consortium. In addition to the 8 DH health workers, circle council members, journalists, chairs of Community Health Associations (Associations de santé communautaire), and Technical Directors of CSCom (Directeurs techniques de centres), participated in a 3-day PBF training session in each DH. The number of trainees and the duration of training was considered insufficient to understand the principles and functioning of the PBF project, given its complexity and the need to adapt it to each context. DHMT members were the most knowledgeable about the different contours of the intervention. There was no regular meeting to inform stakeholders on the implementation of PBF (*Readiness for Implementation/Access to Knowledge and Information*).

According to the respondents, the development of the results plans started during training and was finalised by the human resources manager, the MCD, and the PBF focal point. In addition, except for those who participated in the training, health workers did not receive a document explaining PBF. Some health workers got information on PBF through those who participated. No poster or social marketing strategy for staff and users was used during PBF implementation:

“*Users are not aware that there is a new system called PBF at the DH level”* [PBF Focal Point, Case 1].

Although respondents did not mention measures to combat drug stockouts, PBF would have pushed DHs to better comply with the national drug supply plan. In addition, the vast majority of respondents did not believe that PBF brought anything new with respect to standards and procedures. Health workers did not perceive PBF as a new policy; rather they viewed it as a motivator to do their job better (*Implementation Climate/Compatibility*). In addition, the PBF implementing agency did not provide DHMT members with financial or logistical resources in time to enable for the performance of the indicator verification activities (*Readiness for Implementation*/ *Available resources*):

“*There are logistical problems in conducting supervision... we only have one vehicle [to do this]”* [District Health Manager, Case 2]. 

Finally, the populations apparently were not informed about the implementation of PBF in DHs.

### Characteristics of Individuals (Health Workers and Other Employees) 

The majority of respondents were supportive and enthusiastic about PBF principles (*Knowledge and beliefs about the intervention of the PBF*). According to them, PBF was different from other projects because it improved healthcare quality and patients’ satisfaction, by motivating health workers through individual incentives and investment subsidies for DHs. Although they linked PBF’s intervention to the values of a job well done. Some respondents mentioned the potential negative effects of PBF: (*i*) the lack of motivation of some members of the healthcare team; (*ii*) the competitive spirit leading to individualism and its negative consequences on teamwork; and (*iii*) the excessive increase in diagnostics related to PBF indicators. Respondents believed that there was a problem of ownership of PBF at the regional and national levels, which posed problems at the local level. According to them, the designed project did not allow for the involvement of all the actors who administered healthcare in DHs (*Knowledge and beliefs about the intervention of the PBF*).

In Mali, the health sector is governed by procedures and standards that are regulated by the MSHP and its decentralised structures. This regulation is not always optimal due to a lack of resources (human, material, and financial). However, during the time of the interviews, the vast majority of health workers said they had become more motivated since the onset of PBF (*Self-efficacy*):

“*Without PBF we are paid, and now with PBF we are even more motivated […]. So it becomes a double motivation, in addition to our salary, we will have a motivation of PBF”* [PBF focal point, Case 1]. 

Some respondents complained that they gradually lost the motivation they had at the beginning because of the delays in evaluating the results and in bonus payments (*Individual Stage of Change*). Those involved in care services outside the maternity hospital did not feel committed to implementing PBF because the indicators chosen did not allow them to be involved (*Individual Identification with Organisation*).

### Process for Implementing the Performance-Based Financing Intervention 

The pilot project of PBF, which included in the World Bank-funded SRHP, was supposed to start in 2011. However, it did not start until 2016 and ended 8 months later, without any modification of the initial design (*Executing*). Its implementation was made hastily so as not to lose the funding obtained from the World Bank. During the implementation of PBF at local level, several problems emerged: limited knowledge, insufficient involvement of actors, poor local ownership, and a delay in the definition of the results plans and the signing of the contracts:

*“It’s when time has ran out that you could at last do something quickly, and everything was rushed... and here we are, there’s a lot of problems”* [District Health Manager, Case 1].

The development of the results plans faced multiple challenges in several DHs. In DH 3, the PBF contract was signed on time. While in DH 1 and 2, the lack of leadership from the lead manager (MCD) caused delays in contract signing (*Planning*). Once contracts were signed, MCD were expected to describe the tasks to be included in each agent’s engagement form. This sheet was supposed to allow each health worker to be informed of MCD expectations. This sheet allowed MCD to evaluate health workers (*Planning*). Once completed, individual engagement forms were distributed, but several agents had not yet signed them:

*“Here, our individual engagement contracts have not been signed (4 months after starting the PBF)”* [Nurse, Case 2]. 

Health workers in DH 3 signed their individual engagement forms on time, and posted them in DH offices or rooms. DH 3 had already implemented the forms because they had already participated in a previous PBF pilot project (*Planning*). The appointment by the MCDs of a PBF focal point facilitated the implementation of PBF in DHs (*Engaging/Formally Appointed Internal Implementation Leaders*). In addition, the implementing agency provided 10 technical assistant experts in PBF (ie, 5 national and 5 international experts) for assisting with PBF implementation (*Engaging/External Change Agents*). However, respondents believed that resources (financial, material, and logistic) had not been sufficiently made available to these experts to fulfil their role:

 “*He (PBF expert) did not receive any equipment, desk, seats, machine; and a vehicle had even been requested for supervision: nothing came” *[PBF focal point, Case 2]. 

Under the PBF contract, the MCD supervised and evaluated the heads of each DH unit. Meanwhile, a team from the DRS evaluated the MCD. Subsequently, each DH head of unit supervised and evaluated the agents under their responsibility (*Reflecting and Evaluating*). No evaluation was done at the time of our visit to the DHs. For respondents, PBF improved health facilities supervision at the peripheral level. However, during implementation, frequent monitoring and evaluation required by PBF was not normally done (*Reflecting and Evaluating*). Despite the fact that qualified staff were trained to perform this task, informants considered the verification activities to be one of the most complicated tasks of the PBF project (10 days/month). In addition, according to some health workers, the fact that many of them were absent for 10 days a month for supervision would affect the quality of healthcare offered in DHs. Thus, the verification component of PBF was perceived as a potential disruptor of DHs functioning (*Reflecting and Evaluating*). New support tools (attendance book, register, and retro-information forms in the evacuation-reference system) were put in place for PBF implementation. They made it possible to improve case notification (eg, the systematic use of feedback sheets improved the evacuation reference system):

*“Before PBF, evacuation reference cases were recorded, but no feedback sheet was available. It was by phone that the Directeurs techniques de centres of the CSCom from which the patient came, was given the feedback. Now it’s recorded and it’s even posted the file as it goes along, and it’s systematic”* [Nurse, Case 1]. 

The staff attendance book was implemented to provide information on the first and last name, qualification, arrival and departure time, and signature of the health worker. At first this register was filled in regularly. But after 3 months without the completion of any verification or the payment of any bonus, the vast majority of health workers only occasionally filled the attendance book, thereby questioning the project’s credibility:

*“But after ... 3 months, no bonus, people started asking questions, is that [project] serious?”* [PBF Focal Point, Case 2].

## Discussion

Our study is the first independent study on the implementation of PBF in Mali; and one of the few studies in Africa on the implementation of PBF at the DH level. To our knowledge, it is the only PBF study in French-speaking West Africa that focuses on the DH context. Our analysis of the implementation of PBF at DH level in Mali revealed several challenges related to the design and implementation of the intervention. Respondents adhered to the principle of PBF intervention, yet they felt it did not bring anything new. The study raised several constraints that may have hindered the implementation of PBF within DH. At the political level, the decisions made by national actors, mindful of the limitations of the PBF pilot project, had a considerable influence. The PBF intervention was one of the sub-components of a larger programme – the SRHP, which aimed to increase the use of reproductive health services in Mali. Despite the fact that decision-makers and the implementing agency at the central level knew that the design of the pilot project was inadequate, and despite the short time for implementation; they insisted on going ahead with the intervention so as not to lose World Bank funding.

This further illustrates the fact that, in some instances, decisions to implement PBF pilot projects in low- and middle-income countries depend on donors and the availability of funding, rather than national actors. The results of several studies highlight that there is a lack of ownership of PBF pilot projects by national actors in Africa.^7,29,30,31^ This absence is possibly due to the pilot form, which involves temporal and geographical constraints and external funding, portraying PBF as a vertical type intervention while being presented as a levee for health system reform.^32^ The lack of national ownership is, therefore, not specific to PBF; it is a recurring problem in Africa for any large intervention encouraged by international donors.^33^

In addition, our analysis of PBF implementation in Mali highlighted specific problems in terms of ownership by decentralised-level actors (DRS and MCD), especially in terms of project design. They were not involved in the process of choosing performance indicators that mobilise subsidies. The choice of indicators matched the donors’ priorities, although they were certainly discussed and validated by the national authorities. Our results are similar to those of other studies in Benin and Rwanda^29,34^ which showed that the indicators chosen during the implementation of PBF in Africa often met donors’ priorities.

At the macro-organisational level, the lack of participation of some health workers in the DH in the definition of the results plans, and the lack of information on the functioning of PBF caused significant problems in terms of health workers’ commitment. The introduction of new tools had positive effects on patient notification. The ratio between the number of indicators selected only in maternal and child health, and the number of health workers in a DH (about 50) seemed insufficient to involve all staff. Some people estimate that at least 15 to 20 quantitative indicators in health facilities are needed when implementing PBF, otherwise the focus would be on some services to the detriment of others.^1^ The choices made in Mali thus differed significantly from standards about PBF project design.

The results plans were made during the first training session. The vast majority of DH agents did not participate in defining the objectives of such plans. Thus, it would be difficult for DHs to achieve the performance objectives set in the results plans that had not been defined by the majority health workers. Defining the objectives of the results plans would benefit from a participatory process. A similar hasty selection of the objectives of results plans was observed in Uganda.^11^

As several other studies illustrated,^11,12,13,35,36^ health workers’ lack of information and knowledge of PBF led to their low commitment in implementation. Conversely, given their strong involvement in the smooth running of PBF, DHMT members had a better understanding of the intervention. Several other studies showed unequal knowledge of PBF between the main actors and the remaining of the workforce.^12,36^ Other studies indicated that involvement in the design and information of all local actors were key to the success of PBF, as these factors determine their commitment to reform.^1,11,29,36^ Finally, the new tools and methods of remuneration introduced by the PBF programme appeared to yield mixed results. Our qualitative data indicates that there was insufficient focus on the governance and financial management of DHs. Financial mismanagement of DHs reportedly caused distrust of health workers over fairness in allocating PBF bonus payments. Several studies highlighted health workers’ feeling of unfairness as to the ways PBF bonus payments were distributed.^29,37^ With respect to the new tools introduced by PBF, systematic feedback sheets helped to improve the evacuation reference system. The same observation was made in a study in Rwanda.^35^

At the micro-organisational level, when PBF was implemented, most health workers expressed enthusiasm. The same enthusiasm and positive effect of the arrival of PBF on health workers has been observed in several other studies in Benin, Burkina Faso, and Malawi.^16,29,36^ However, local actors’ enthusiasm is not the only ingredient for project success. Since the PBF is a complex intervention in an already complex and highly hierarchical health system, the leadership of DH managers emerged as a determining factor in the success or otherwise of the PBF intervention. The lack of engagement of MCDs in some DHs caused recurring delays and malfunctions during the implementation of PBF. When implementing PBF, the leadership capacity of DHs’ managers was considered a factor influencing the implementation of the PBF in DHs. Several other studies in Benin, Rwanda, and Sierra Leone^14,38,39^ confirmed this statement. Indeed, their own understanding of the intervention and their commitment play a key role in the implementation. These are the main actors that can influence the motivation of health workers.

Our research has several limitations. First, the CFIR analysis framework with its different taxonomies, dimensions, and constructs enabled to identify all the contours of the implementation of the PBF project. However, during the analysis of the results, integrating of themes that emerged inductively proved to be complex. During the analysis, some constructs and sub-constructs of different dimensions provided information on the same contents (for example: the sub construct *Relative Advantage* of the dimension *Intervention Characteristic* and the sub-built *Implementation Climate/Relative Priority* of the *Inner Setting* dimension).

The second limitation of the CFIR is that it does not allow for accounting for the complexity and dynamics of interventions, because the constructs used are very descriptive and systematic, but somewhat too linear. The results of the study allowed us to spot some expected and unexpected effects of the PBF intervention in DHs. We highlight some of the most salient effects — as perceived by informants and effectively observed by the first author of this manuscript — in Table 3. A specific qualitative study on the effects of PBF in DHs is necessary to specify the preliminary effects we observed during our study.

**Table 3:** Expected and Unintended Effects of the PBF Pilot Project in DHs

**Expected Effects**	**Unexpected Effects**
• Establishment of results plans in DHs • Increase in patient notification of patients attended to in DHs • The implementation of the PBF pilot scheme addressed some of the malfunctions of the management bodies • The systematic use of feedback sheets improved the evacuation-reference system • The establishment of new support tools (attendance book, register, feedback sheets in the evacuation-reference system) made it possible to strengthen the health system • The introduction of the attendance book at the beginning of PBF implementation in DHs allowed health workers to be more timely, • Hygiene in DHs improved	• The low number of PBF quantity indicators did not allow all stakeholders to be involved in the intervention • The delay in verification and evaluation within DHs may have discouraged health workers • As a consequence of the delay in verifying and paying premiums, health workers no longer filled in the attendance register correctly as they had done at the beginning of the PBF intervention

Abbreviations: PBF, performance-based financing; DHs, district hospitals.

The CFIR is not designed to establish the correlation/causality links (between implantation components/processes/internal and external context on the one hand; and effects on the other hand). The study of the causal links between the contextual factors of the PBF pilot project and the effects of this project would merit a full and specific study. A theoretical framework that would be adapted to this type of study is the contribution analysis method by Mayne.^40^

Three additional limitations can be drawn from our study. Data collection took place when most DHs had only started PBF for 3 or 4 months. Therefore, apart from the main actors implementing PBF in DHs, other actors’ discourse and perceptions about the intervention were limited. Furthermore, patients have not been included in the interviewed persons.

Lastly, our study is cross-sectional: it does not allow us to draw a conclusion on the evolution of the process of implementing PBF at DH level. Nevertheless, our study enabled us to review the quality of the PBF intervention model, the implementation process, the ownership of PBF at the DH level, and to identify lessons learned and areas of improvement in DHs. Further longitudinal research may allow for a more robust study of the process of implementing PBF at the DH level. It will also support other health reforms that can be implemented.

## Conclusion

Our qualitative research at the DH level in Mali allowed us to highlight local actors’ representations of PBF, and to understand how the context and the norms and values influence its implementation. The PBF project offered an opportunity to improve the quality of care provided to the population through the motivation of health personnel in Mali. Respondents adhered to the principles of PBF but argued that it brought nothing new. Several challenges that could hinder the successful implementation of PBF within DHs were raised. We offer some recommendations to improve implementation. First of all, in order to mitigate the information problems with PBF, it would be useful to have effective communication at the start of the project to allow a better local appropriation. Secondly, the number of quantitative indicators chosen should be sufficient to allow all actors to be involved in project implementation. Finally, the logistical and financial resources should be made available to the DHMT members so that the quantitative and qualitative verifications are carried out on time. From its conception and during the implementation of PBF in DHs, it is necessary to consider the factors that can influence the implementation of such a complex intervention. Future qualitative studies are needed to understand how PBF affects the leadership and management of managers in DHs, and how PBF affects the involvement of DHMTs’ members in the supervision of the centres of health at the peripheral level during implementation. Studies on the distribution of bonus payments in DHs and how it affects the motivation of health workers are also needed.

## Acknowledgements

This work has been supported by several donors including the International Development Research Centre. The research was carried out as part of a larger programme on “*Results-based financing for equitable access to maternal and child health care in Mali and Burkina Faso.*” It is part of the 7-year Innovating for Maternal and Child Health in Africa. This research program was implemented by a team of NGOs based in both countries, and researchers from the University of Montreal. In Mali, this team was composed of researchers and research assistants from the NGO MISELI led by Laurence Touré, who is the Principal Researcher of the programme. We would also like to thank all the actors and organisations at national, regional, and local levels who participated and/or facilitated the conduct of this research. We would particularly like to thank Laurence Touré for her advice and support during the conduct of this research. Finally, we would like to thank Heather Hickey for her help in translating the article from French into English.

## Ethical issues

The research protocol has been validated by the ethics committee of the National Institute of Public Health Research (INRSP) of Mali (N°24/2015/CE-INRSP). All participants were informed in detail about the objectives of our research. We obtained the free and informed consent from all the participants.

## Competing interests

Authors declare that they have no competing interests.

## Authors’ contributions

The research protocol was developed by VR with the contribution of AC and LG. The collection tools were developed by TZ and AC with the contribution of VR and LG. TZ organised the data collection in district hospitals (DHs). The analyses were carried out by TZ with the contribution of VR, LG, and AC. TZ wrote the first version of the article. All authors have read, improved, and approved the final version of the article.

## Authors’ affiliations

^1^CEPED (UMR 196), Institut de Recherche pour le Développement, ERL INSERM SAGESUD, École doctorale Pierre Louis de santé publique, Université Sorbonne Paris Cité, Paris, France. ^2^Miseli Research NGO, Bamako, Mali. ^3^Department of Social and Preventive Medicine, University of Montreal, Montreal, QC, Canada. ^4^Public Health Research Institute, University of Montreal, Montreal, QC, Canada. ^5^CESSMA (UMR 245), Institut de Recherche pour le Développement, Université Sorbonne Paris Cité, Paris, France. ^6^Faculty of Medicine and Odonto-Stomatology, Université des Sciences, des Techniques et des Technologies, Bamako, Mali. ^7^CEPED (UMR 196), Institut de Recherche pour le Développement, ERL INSERM SAGESUD, Université Sorbonne Paris Cité, Paris, France.

## Supplementary files


Supplementary file 1 contains Figure S1 and Tables S1-S5.

## Key messages

**Implications for policy makers**

- Involving decentralised actors in the process of choosing performance-based financing (PBF) indicators is essential for its success because it allows for better ownership of the intervention at the local level.
- Factors that may influence the leadership and management capacity of district hospitals (DHs) need to be taken into account at the start of PBF as this supports its implementation.
- The limited number of indicators paid by PBF in DHs can have negative effects on its implementation, as it may reduce the involvement of all health workers in achieving results.

**Implications for public**

The main objective of performance-based financing (PBF) is to improve the quality of care provided to the population through the motivation of health workers. Our study on the implementation of PBF in district hospitals (DHs) in Mali illustrates that the leadership capacity of the first DH manager is a factor that influences the implementation and the ownership of PBF in DHs. In addition to the contextual factors, our study identified some effects of PBF implementation in Mali. Our study showed that the establishment of new support tools (attendance book, register, feedback sheets in the evacuation-reference system) made it possible to strengthen the health system.
