# Supplementary material for: Stakeholder Perceptions and Context of the Implementation of Performance-Based Financing in District Hospitals in Mali
Source: Int J Health Policy Manag. 2019 Jun 30;8(10):583–92. doi: 10.15171/ijhpm.2019.45 (PMC6819625; doi:10.15171/ijhpm.2019.45)
Supplement: Supplementary file 1 — contains Figure S1 and Tables S1-S5. [file ijhpm-8-583-s001.pdf]

Supplementary File 1

Table S1. List and Unit Cost of Quantitative Indicators at DHs Levels

**Indicators/Services PCA**	**Unit cost in CFA Franc**
Number of complications managed at the time of delivery (including caesarean sections...)	13 227
Number of cases referred correctly handled and counter-referred	992
Number of cases of complicated malaria in children (0-5 years)	992

Source: Procedure Manual in Mali (2016).

Abbreviations : Complementary Activity Package (Paquet Complémentaire d'Activité), PCA.

Table S2. Weighting of qualitative indicators for CSCom and DHs

**Headings**	**Weight**
Resources and processes: indicators on human resources, infrastructure, patient relations, functionality, hygiene, governance, role of the ASACO, etc.	30
Clinical indicators: availability of essential medicines, compliance with quality standards, etc.	50
User satisfaction	20
**Total**	100

Source: Procedure Manual in Mali (2016)

Abbreviations : Community Health Association (Association de Santé Communautaire), ASACO ; Community Health Center (Centre de santé communautaire), CSCom; District hopital, DH.

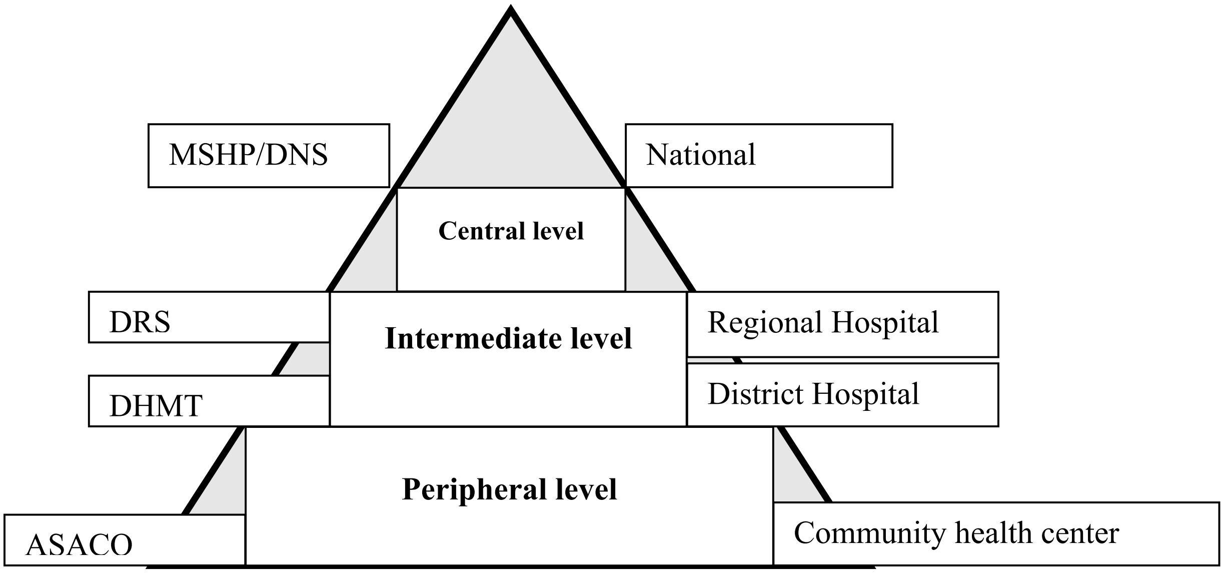

Figure S1. Health Pyramid of Mali.

Abbreviations : Community Health Association (Association de Santé Communautaire), ASACO ; District Health Management Team (Membre de l'Equipe Cadre du District), DHMT; National Health Directorate (Direction Nationale de la Santé), DNS ; Regional Health Directorate (Direction Régionale de la santé), DRS ; Ministry of Health and Public Hygiene (Ministère de la Santé et de l'Hygiène Publique), MSHP.

Table S3. Socio-demographic and health characteristics in Mali and the Koulikoro region

Characteristics	Country	Koulikoro Region
Population [1] (2017 estimate)	18.786.996	3.146.696 (16%)
Surface area	1 241 238 km²	90 120 km²
% of the population residing in rural areas	75.5 %	94.5%
Rate of urbanization	22.5 %	5.5%
Incidence of poverty[2] (proportion of the population considered poor)	46.8 (2016)	51.5 (2016)
Number of health districts	65	10
Number of district hospitals	65	10
Number of CSCom	1.296	205
Ratio of health personnel (doctor, midwife and nurse) per 10,000 inhabitants	5.2	3.7
Female of childbearing age (15-49 years)	3.901.903 (22 % of the total population)	653.543
Child under 5 years of age	3.015.107 (17 % of the total population)	505.010
Morbidity rate (%) [3]	27.3 (2018)	21.7 (2018)
Proportion of the population living within 5 km of a health facility (%)	76.2 (2016)	67.6 (2016)
Proportion of population living more than 5 km from a health facility (%)[4]	23.8	32.4
Proportion of population who consulted in health facilities (%) [4]	82.7	94.9

Table S4. Description of the different constructions of the CFIR framework and justification for the choice of those not selected

Construct	Short Description	Justification of unsuccessful constructs
**1. INTERVENTION CHARACTERISTICS**		
A. **Intervention Source**	The actors' perception of the origin of the intervention. Has it been developed internally or externally? Why was it developed?	
B. **Evidence Strength & Quality**	Stakeholders' perceptions of the quality and validity of evidence that the intervention will achieve its intended outcomes (e.g., intervention theory).	
C. **Relative Advantage**	Stakeholders' perception of the benefit of implementing the intervention compared to other forms of intervention.	
D. **Adaptability**	The degree to which the intervention can be adapted or reinvented to meet local needs	
**E. Triability**	The ability to test the intervention on a small scale within the organization, and to be able to change (or cancel the implementation) if justified.	
F. **Complexity**	The perceived difficulty of implementing the intervention, particularly in terms of duration, scope, radicality, level of disruption, centrality, and complexity; and the number of steps required to implement it.	

G. **Design Quality & Packaging**	Perceived excellence in the way the intervention is presented, assembled, and designed	
**H. Cost**	The costs of the intervention and the costs associated with its implementation, including investment, procurement, and opportunity costs.	
**2. OUTER SETTING**	**Short Description**	**Justification of unsuccessful constructs**
A. **Patient Needs & Resources**	The degree to which patients' needs, as well as the barriers and facilitators to meet these needs, are known and prioritized by the organization.	
B. **Cosmopolitanism**	The degree to which the organization is networked with external organizations.	
C. **Peer Pressure**	Pressure to implement the intervention, usually because other major organizations have already implemented it or because there is a desire to have a competitive advantage.	We did not have any data on this structure.
D. **External Policy & Incentives**	A broad framework that includes external strategies for disseminating the intervention, including policies and regulations (central government entity or other), external mandates, recommendations and guidelines, and public reporting.	

3.INNER SETTING	Short Description	Justification of unsuccessful constructs
A. **Structural Characteristics**	The social architecture, age, maturity, and size of the organization.	
B. **Networks & Communications**	The nature and quality of social networks, and formal and informal communications in the organization.	
C. **Culture**	The norms, values, and foundations of the organization.	
D. **Implementation Climate**	The capacity to absorb change, the shared responsiveness of those involved in the intervention, and the extent to which the use of the intervention will be rewarded, supported, and expected within the organization.	
1.**Tension for Change**	The degree to which stakeholders perceive the current situation as intolerable or in need of change.	
2.**Compatibility**	The degree of correspondence between the meaning and values of the intervention and the norms, values, and perceived needs of individuals. The degree of correspondence between the intervention and work flow and existing systems.	
3.**Relative Priority**	The shared perception of individuals about the importance of implementing the intervention in the organization.	This construction is already considered in our case in the "Relative Advantage."

4. **Organizational Incentives & Rewards**	Extrinsic incentives such as rewards, performance appraisals, promotions, and salary increases; and less tangible incentives such as improved stature or respect.	The constructed "Organizational Incentive and Reward" has not been considered because the PBF itself is already considered as an incentive and reward with bonuses.
5. **Goals and Feedback**	The degree to which objectives are clearly communicated and submitted to staff and the alignment of feedback with objectives.	
6. **Learning Climate**	A climate in which: a) leaders express their own fallibility and need for help and contribution from team members; b) team members feel that they are essential, valued, and competent partners in the change process; c) individuals feel psychologically safe to try new methods; and d) there is sufficient time and space for reflection and evaluation.	We were unable to obtain any information about this structure.
E. **Readiness for Implementation**	Tangible and immediate indicators of organizational commitment to implement the intervention.	
1. **Leadership Engagement**	The commitment, involvement, and responsibility of leaders and managers for implementation.	
2. **Available Resources**	The amount of resources devoted to implementation and ongoing operations, including money, training, education, physical space, and time.	

**3. Access to Knowledge & Information**	Easy access to digestible information and knowledge about the intervention and how to incorporate it into work tasks.	
**4. CHARACTERISTICS OF INDIVIDUALS**	**Short Description**	**Justification of unsuccessful constructs**
A. **Knowledge & Beliefs about the Intervention**	The attitudes of individuals and the values attributed to the intervention, as well as knowledge of the facts, truths, and principles related to the intervention.	
**B. Self-efficacy**	Individuals' belief in their own ability to execute action plans to achieve implementation goals.	
**C. Individual Stage of Change**	The phase an individual is in as he or she progresses towards better use of the intervention (e.g., qualified, enthusiastic, and supported).	
**D. Individual Identification with Organization**	A broad construct related to how individuals perceive the organization and their relationship and the degree of engagement with it.	
**E. Other Personal Attributes**	A broad construct to include other personal characteristics such as ambiguity tolerance, intellectual ability, motivation, values, competence, ability, and learning style.	

5. PROCESS	Short Description	Justification of unsuccessful constructs
**A. Planning**	The degree to which plans, methods, and tasks for implementing an intervention are developed in advance, and the quality of these methods.	
**B. Engaging**	Attract and involve appropriate people in the implementation and use of the intervention through a combined strategy of social marketing, education, role modeling, training, and other similar activities.	
1. **Opinion Leaders**	Individuals in an organization who have a formal or informal influence on the attitudes and beliefs of their colleagues regarding the implementation of the intervention.	
**2. Formally Appointed Internal Implementation Leaders**	Individuals in the organization who have been formally appointed responsible for the implementation of an intervention as coordinators, project managers, team leaders, or other similar roles.	
**3. Champions**	People who are dedicated to supporting, marketing, and conducting the implementation, to overcoming the indifference or resistance that the intervention can cause in an organization.	We were unable to obtain any information about this structure because the implementation time was not long enough to allow the emergence of champions.

**4. External Change Agents**	People who are affiliated with an external entity that formally influences or facilitates decisions related to an intervention.	
**5. Intervention Participants**	People who are directly or indirectly involved in the intervention (e.g., health workers, patients)	
**C. Executing**	The implementation in accordance with the plan.	
**D. Reflecting & Evaluating**	Quantitative or qualitative evaluations on progress and quality of implementation with personal and regular team debriefings on progress and experience.	
**E. Evolution**	Changes made to the intervention over time to improve it.	We did not get an answer for this construct because our survey was conducted at the beginning of the implementation. Therefore, no changes had yet been made at the local level to improve the implementation of the PBF intervention.

Appendix S5. Characteristics of the three health districts and their DHs

Characteristics	District 1	District 2	District 3
Population [5]	210.611 hbts (RGPH 2009)	50.039 hbts (RGPH 2009)	488.937 hbts (RGPH 2009)
Surface area	7 260 km²	1 118 km²	12 794 km2
Distance from the capital where the 3rd reference level is located (University Hospital and national hospitals)	60 Km	80 Km	165 Km
Incidence of poverty [6] (proportion of the population considered poor)	35-40%	31-35%	44-48%
Wealth and savings	-Sand and gravel mining, agriculture, livestock and fisheries are the main economic activities,<br>-Existence of some industrial units.	-Agriculture and livestock are the main activities,<br>-Crafts and trade also developed.	-Presence of rich and fertile lands,<br>-90% of the population is agropastoral,<br>-Rural economy based largely on cotton cultivation.
Number of district hospitals	1	1	1
Total health staff in the district hospital	76	35	54
Number of physicians	7	9	4

Number of CSCom [7]	21	16	24
Number of localities [5]	249	45	352

Footnote

1. INSTAT. Analysis report of the modular and permanent household survey (EMOP). National Institute of Statistics; 2017:75.

2. INSTAT. Analysis report of the modular and permanent household survey (EMOP). National Institute of Statistics; 2017:105.

3. INSTAT. Analysis report of the modular and permanent household survey (EMOP)/Health, employment, food security and household consumption expenditure. National Institute of Statistics; 2018:51.

4. INSTAT. Analysis report of the modular and permanent household survey (EMOP)/accessibility to care, population assessment of priority actions to be undertaken and household consumption expenditure. National Institute of Statistics; 2016:50.

5. INSTAT. General Population and Housing Census (GPHC). Institut National de la Statistique; 2009:309.

6. INSTAT. Mapping poverty and the Millennium Development Goals (MDGs) in Mali/ (RGPH-2009). National Institute of Statistics; 2012:84.

7. Human Resources Directorate of the Health, Social Development and Family Promotion Sector. Statistical Yearbook 2016 of human resources in the health, social development and family promotion sector. Human Resources Directorate of the Health, Social Development
